# Novel Diagnostic and Prognostic Tools for Lung Cancer Cachexia: Based on Nutritional and Inflammatory Status

**DOI:** 10.3389/fonc.2022.890745

**Published:** 2022-07-11

**Authors:** Chen-An Liu, Qi Zhang, Guo-Tian Ruan, Liu-Yi Shen, Hai-Lun Xie, Tong Liu, Meng Tang, Xi Zhang, Ming Yang, Chun-Lei Hu, Kang-Ping Zhang, Xiao-Yue Liu, Han-Ping Shi

**Affiliations:** ^1^ Department of Gastrointestinal Surgery, Beijing Shijitan Hospital, Capital Medical University, Beijing, China; ^2^ Department of Clinical Nutrition, Beijing Shijitan Hospital, Capital Medical University, Beijing, China; ^3^ Key Laboratory of Cancer Food for Special Medical Purposes (FSMP) for State Market Regulation, Beijing, China; ^4^ Department of Pathology and Shanxi Key Laboratory of Carcinogenesis and Translational Research of Esophageal Cancer, Shanxi Medical University, Taiyuan, China

**Keywords:** lung cancer, cachexia, prognosis, nutrition, Inflammation, nomogram

## Abstract

**Background:**

Cachexia is one of the most common complications affecting lung cancer patients that seriously affects their quality-of-life and survival time. This study aimed to analyze the predictors and prognostic factors of lung cancer cachexia as well as to develop a convenient and accurate clinical prediction tool for oncologists.

**Methods:**

In this multicenter cohort study, 4022 patients with lung cancer were retrospectively analyzed. The patients were randomly categorized into training and verification sets (7:3 ratio). Univariate and multivariate logistic regression analyses were performed to determine the risk factors of cachexia in patients with lung cancer. Cox regression analysis was applied to determine independent prognostic factors in the patients with lung cancer cachexia. Meanwhile, two nomograms were established and evaluated by time-dependent receiver operating characteristic curve, calibration curve, and decision curve analysis (DCA).

**Results:**

Stage, serum albumin, ALI, anemia, and surgery were independent risk factors for cachexia in patients with lung cancer. Patients with lung cancer cachexia have a shorter survival time. Sex, stage, serum albumin, ALI, KPS score, and surgery served as independent prognostic factors for patients with lung cancer cachexia. The area under the curves (AUCs) of diagnostic nomogram in the training and validation sets were 0.702 and 0.688, respectively, the AUCs of prognostic nomogram in the training set for 1-, 3-, and 5-year were 0.70, 0.72, and 0.75, respectively, while in the validation set the AUCs were 0.71, 0.75, and 0.79, respectively. The calibration curves and DCA of the two nomograms were consistent and the clinical benefit rate was high.

**Conclusion:**

Cachexia brings an additional economic burden and worsens the prognosis of lung cancer patients. The two nomograms can accurately screen and predict the probability of occurrence of cachexia in lung cancer and the prognosis of patients with lung cancer cachexia, and guide clinical work.

## Introduction

As per the latest statistical report on cancer, lung cancer remains the top killer, threatening human public health safety and being the primary cause of malignant tumor death, accounting for approximately 21% of all deaths from cancer. Although several prevention and treatment strategies have been developed so far for lung cancer treatment, such as smoking cessation, surgery, radiotherapy, chemotherapy, targeted therapy, and immunotherapy, the American Cancer Society estimates that approximately 350 people will die of lung cancer every day in 2022 (1). However, considering the several death factors associated with lung cancer, approximately 20% of the patients with advanced lung cancer die due to nutritional issues such as the continuous reduction of skeletal muscle and body fat (2). This metabolic disorder has also gained attention in recent years and is referred to as cancer cachexia.

Cancer cachexia is a multifactorial syndrome characterized by ongoing loss of skeletal muscle mass, with or without the loss of fat mass, that cannot be completely reversed through routine nutritional support treatment, gradually leading to functional impairment (3). Patients with cancer cachexia often experience symptoms such as anorexia, satiety, decreased body mass index (BMI), muscle atrophy, fatigue, anemia, edema, and hypoproteinemia. In addition, owing to the secretion disorder of inflammatory cells and immune cells, inflammatory activation, proteolysis, autophagy, and lipolysis, the occurrence of cancer cachexia is often accompanied by the abnormalities of endocrine, metabolic, and central nervous systems, as well as the destruction of the myocardium, adipose tissues, and liver (4). The evolution of cachexia of cancer cachexia can be divided into three distinct stages: precachexia, cachexia, and refractory cachexia (5). Past studies have demonstrated that, with the development of this disease, cancer cachexia can seriously affect the quality-of-life, reduce chemotherapy response, increase chemotherapy toxicity, and even affect the survival of patients with advanced cancer (6). Especially, patients at the final stage present an irreversible catabolic state, show little response to anti-cancer treatment, and have an expected survival time of <3 months (7).

The consensus on the definition of cancer cachexia has been accepted, and it is recognized that it predicts a poor prognosis. Unfortunately, patients often enter the advanced stage of cachexia even before oncologists can identify them. This may be closely related to the heterogeneity of clinical presentation (8), the untimely monitoring of weight and body composition (9), and the confusion of sarcopenic obesity (10). Therefore, the prevention, identification, intervention, and prognosis of cancer cachexia remain challenging. Although some studies have attempted to determine biomarkers or predictive factors useful for the diagnosis and prognosis of lung cancer patients with cachexia and have developed several models to assess early cachexia and predict its prognosis (11–13). However, their use in clinical practice of lung cancer is not established and eventually identification of early cancer cachexia is yet a major problem in clinical practice.

In the present study, we aim to assess the economic burden, screen predictors and prognostic factors of patients with lung cancer cachexia by using our multicenter cohort data. In addition, we want to establish and validate a nomogram for screening the presence of cachexia in lung cancer patients and predicting the prognosis of lung cancer cachexia patients. Compared with the traditional diagnostic criteria for cachexia, the nomogram adopts some of our previous findings (14, 15), and integrates more easily obtained laboratory indicators and patient tumor characteristics as predictors to provide basis for clinical diagnosis and treatment of this disease.

## Materials and Methods

### Study Population

The patients’ data were sourced from a large-scale, multicenter, prospective, observational cohort study titled the Investigation on Nutrition Status and its Clinical Outcome of Common Cancers (INSCOC), which was registered at chictr.org.cn (registration number: ChiCTR1800020329). The data on 12,792 patients in this project was sourced from more than 40 clinical centers across China; these patients were diagnosed with malignant tumors by pathological examinations from June 2012 to December 2019. In INSCOC, we included first-time hospitalized patients of age ≥18 who voluntarily participated in this study and received antitumor treatment, and excluded readmission patients who had participated in this study, those who had received organ transplantation, those who had received antineoplastic therapy in the past, those diagnosed with AIDS infection, pregnant patients, and intensive care unit (ICU) patients. This retrospective study was conducted in adherence to the guidelines specified by the Helsinki Declaration and with approval by the ethics committee of each participating clinical center. All participants provided their signed informed consent. The reporting of the present study conforms to the TRIPOD guidelines (16).

### Data Collection

We retrospectively obtained the clinical and laboratory data from the medical records of patients. (1) The clinical characteristics included the gender, age, BMI index (underweight, <18.5 kg/m^2^, normal, 18.5–23.9 kg/m^2^, overweight, 24.0–27.9 kg/m^2^, obesity, ≥28 kg/m^2^), Patient-Generated Subjective Global Assessment (PG-SGA), Karnofsky Performance Status (KPS), and nutritional risk screening (NRS 2002), (2) Past and personal history (diabetes, hypertension, coronary heart disease, family history of tumor, smoking, and drinking), (3) Tumor characteristics (treatment and the tumor stage), (4) Laboratory indices included serum creatinine (Scr), albumin, neutrophils, lymphocytes, platelets, and hemoglobin, (5) Diagnosis of cachexia and Survival information (survival time and status). All assessments were conducted within 48 h of admission, and the survival status, survival time of patients, were followed up *via* telephone, outpatient service, and other means. In addition, we also collected information on the hospitalization expenses of the patients.

### Diagnosis and Variable Definition

Cancer cachexia was diagnosed through three items, as proposed by Fearon et al. in 2011, as follows: (1) weight loss >5% over the past 6 months (in the absence of simple starvation); or (2) BMI <20 and any degree of weight loss >2%; or (3) appendicular skeletal muscle index consistent with sarcopenia and any degree of weight loss >2% (17). Skeletal muscle depletion was assessed by anthropometry of the upper-middle arm muscle area (men <32 cm^2^, women <18 cm^2^). The tumor stage was defined as per the 8^th^ edition specifications of the AJCC TNM staging system. Based on their different Scr levels, the patients were categorized into low (<71 mmol/L in men or <59 mmol/L in women), normal (71–104 mmol/L in men or 59–85 mmol/L in women), and high (>104 mmol/L in men or >85 mmol/L in women) Scr groups (18). Anemia was defined as hemoglobin level <120 g/L in men or hemoglobin level <110 g/L in women. Advanced lung cancer inflammation index (ALI) was calculated using the following formula: BMI × albumin (g/dL)/NLR. Where NLR is the neutrophil-lymphocyte ratio.

### Study Endpoint and Follow-Up

Our main study objective was to assess the risk factors of cachexia in lung cancer patients as well as the prognostic factors of patients with lung cancer cachexia. Hence, the overall survival (OS) of the lung cancer patients with cachexia served as the study endpoint. The follow-up time was up to 30 September 2019 or the time of the last contact.

### Statistical Analysis

The continuous variables were expressed as mean ± standard deviation (SD), while the categorical data were expressed in numbers and percentages; the comparison between groups was expressed in the χ2 test or Fisher exact test. We applied the restricted cubic spline regression to evaluate the continuous variable (ALI) as well as to determine its optimal cutoff value. Univariate and multivariate logistic regression analyses were performed to determine the risk factors of cachexia in patients with lung cancer. Meanwhile, Odds Ratio (OR) and 95% confidence interval (95% CI) were calculated, and a nomogram model was established. We performed the Kaplan–Meier (K–M) survival analysis to complete the survival curve and compared it with the results of the log-rank test. Univariate and multivariate Cox model were applied to analyze the prognostic factors. The nomogram model was established based on the prognostic factors, and the Hazard Ratio (HR) and 95% CI were calculated. The prediction performance of the model was evaluated by consistency index (C-index), the prediction coincidence was judged by calibration curve, and the clinical practicability of the model was evaluated by decision curve analysis (DCA). All data were analyzed by R software (R Foundation for Statistical Computing-project.org 4.0.2). The differences were considered to indicate statistical significance at two-sided P < 0.05.

## Results

### Patient Clinical Characteristics

**Figure 1:**
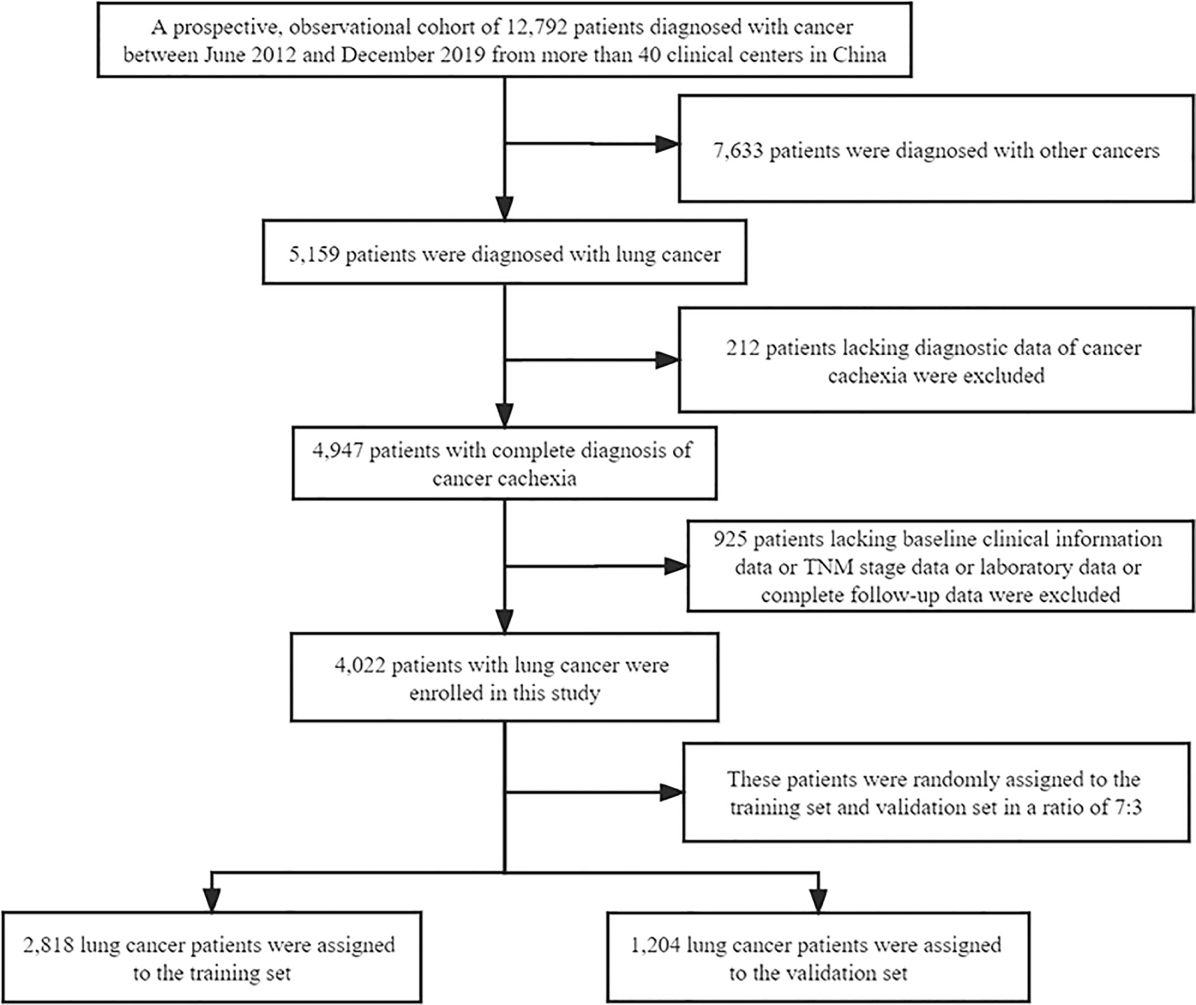
Flow chart of study design.

A total of 4022 patients were enrolled in this study, of which 2818 were included in the training set and 1204 in the validation set. The patients’ baseline clinical characteristics are shown in **Table 1**. The flow chart of patient screening and the study design is depicted in **Figure 1**. The average age of the patients was 60.16 ± 10.00 years, and men accounted for the majority (66.3%) of all patients. In addition, 51.8% of the patients died during the follow-up, and the median OS was 26.73 months (95%: 24.91–28.56). In this study, the incidence of cachexia in patients with lung cancer was 27.9%, which included 61 patients with stage I, 127 patients with stage II, 205 patients with stage III, and 733 patients with stage IV of the disease. In addition, we compared the initial hospitalization expenses of all lung cancer patients with and without cachexia (USD, 3406.72 ± 99.30 vs. 2925.90 ± 59.29). We noted a significant statistical difference between the two groups (**Figure S1**). In **Figure S2**, we employed restricted cubic splines regression to flexibly demonstrate the relationship between ALI and all-cause mortality in patients with lung cancer and specified 34.93 as the cutoff value of ALI.

**Table 1:** Demographic and clinical characteristics of patients diagnosed with lung cancer.

		training set N=2818	validation set N=1204	χ^2^	P
Sex	famale	950 (33.7%)	402 (33.4%)	0.039	0.843
	male	1868 (66.3%)	802 (66.6%)		
Age (years)	≤65	1959 (69.5%)	861 (71.5%)	4.601	0.206
	>65	859 (30.5%)	343 (28.5%)		
Cachexia	no	2020 (71.7%)	876 (72.8%)	0.484	0.487
	yes	798 (28.3%)	328 (27.2%)		
Diabetes	no	2558 (90.8%)	1097 (91.1%)	0.117	0.732
	yes	260 (9.2%)	107 (8.9%)		
Hypertension	no	2273 (80.7%)	965 (80.1%)	0.140	0.708
	yes	545 (19.3%)	239 (19.9%)		
Coronary heart disease	no	2681 (95.1%)	1131 (93.9%)	2.461	0.117
	yes	137 (4.9%)	73 (6.1%)		
Family history	no	2355 (83.6%)	1026 (85.2%)	1.706	0.192
	yes	463 (16.4%)	178 (14.8%)		
Smoke	no	1124 (39.9%)	498 (41.4%)	0.763	0.382
	yes	1694 (60.1%)	706 (58.6%)		
Drinking	no	2153 (76.4%)	907 (75.3%)	0.530	0.467
	yes	665 (23.6%)	297 (24.7%)		
Stage	1/2	704 (25.0%)	289 (24.0%)	0.435	0.510
	3/4	2114 (75.0%)	915 (76.0%)		
Surgery	no	2503 (88.8%)	1083 (90.0%)	1.111	0.292
	yes	315 (11.2%)	121 (10.0%)		
Chemotherapy	no	1065 (37.8%)	475 (39.5%)	0.983	0.322
	yes	1753 (62.2%)	729 (60.5%)		
Radiotherapy	no	2586 (91.8%)	1101 (91.4%)	0.115	0.735
	yes	232 (8.2%)	103 (8.6%)		
KPS	low	181 (6.4%)	78 (7.0%)	0.420	0.517
	high	2637 (93.6%)	1126 (93.0%)		
Scr	low	1338 (47.5%)	593 (49.3%)	2.234	0.327
	normal	1339 (47.5%)	562 (46.7%)		
	high	141 (5.0%)	49 (4.1%)		
Albumin (g/L)	<35	598 (21.2%)	242 (19.9%)	0.961	0.327
	≥35	2220 (78.8%)	962 (80.1%)		
Anemia	no	2077 (73.7%)	893 (74.2%)	0.094	0.759
	yes	741 (26.3%)	311 (25.8%)		
ALI	low	1539 (54.6%)	649 (53.9%)	0.171	0.679
	high	1279 (45.4%)	555 (46.1%)		
Platelet (×10^9^/L)	<100	2690 (95.5%)	1164 (96.3%)	1.624	0.203
	≥100	128 (4.5%)	40 (3.7%)		
BMI	underweight	248 (8.8%)	100 (8.3%)	6.255	0.100
	normal	1572 (55.8%)	722 (60.0%)		
	overweight	824 (28.2%)	318 (26.4%)		
	obesity	174 (6.2%)	64 (5.3%)		
PG SGA	≤3	1016 (36.1%)	446 (37.0%)	0.357	0.550
	>3	1802 (63.9%)	758 (63.0%)		
NRS2002	<3	2111 (74.9%)	902 (74.9%)	0.000	0.997
	≥3	707 (25.1%)	302 (25.1%)		

### Predictors of Cachexia in Patients With Lung Cancer

The clinical characteristics (age, gender), past and personal history (diabetes, hypertension, and coronary heart disease, family history, smoking and drinking history), tumor characteristics (stage, surgery, radiotherapy, chemotherapy), and laboratory indices (Scr, albumin, platelet, anemia, ALI) were included as the analysis of predictors. In univariate logistic regression analysis, age, hypertension, stage, Scr and albumin, ALI, anemia, surgery, and radiotherapy were all associated with cachexia in patients with lung cancer. When these factors were included in multivariate logistic regression analysis, anemia, disease stage, albumin, ALI, and surgery served as independent predictors for cancer cachexia in lung cancer patients (**Table 2**). Anemia (P < 0.001) and advanced stage (III/IV, P = 0.002) acted as the risk factors of cancer cachexia, while high albumin (>35 g/L, P < 0.001), high ALI (>34.93, P < 0.001), and surgery (P < 0.001) serving as the protective factors of cancer cachexia. In order to better demonstrate the effectiveness of these factors, we used a forest map to illustrate the independent predictors (**Figure S3**).

**Table 2:** Logistic regression analysis of risk factors of cancer cachexia in patients with lung cancer.

		Univariate analysis		Multivariate analysis	
		OR (95%CI)	P	OR (95%CI)	P
Age (years)	≤65				
	>65	1.242 (1.043-1.480)	0.015		
Sex	famale	1			
	male	1.024 (0.861-1.218)	0.789		
Diabetes	no				
	yes	1.139 (0.863-1.503)	0.357		
Hypertension	no				
	yes	0.790 (0.637-0.979)	0.032		
Coronary heart disease	no				
	yes	0.762 (0.508-1.142)	0.188		
Family history	no				
	yes	0.936 (0.749-1.171)	0.564		
Smoke	no				
	yes	1.135 (0.959-1.344)	0.140		
Drinking	no				
	yes	1.129 (0.933-1.366)	0.212		
Stage	1/2				
	3/4	2.088 (1.698-2.582)	<0.001	1.450 (1.152-1.825)	0.002
Scr	low	1.365 (1.152-1.618)	<0.001		
	normal	1			
	high	1.955 (1.364-2.804)	<0.001		
Albumin (g/L)	<35				
	≥35	0.424 (0.351-0.512)	<0.001	0.605 (0.526-0.802)	<0.001
ALI	low				
	high	0.369 (0.309-0.440)	<0.001	0.463 (0.384-0.559)	<0.001
Platelet (×10^9^/L)	<100				
	≥100	1.446 (0.998-2.095)	0.051		
Anemia	no				
	yes	2.401 (2.010-2.867)	<0.001	1.785 (1.471-2.166)	<0.001
Surgery	no				
	yes	0.454 (0.332-0.620)	<0.001	0.492 (0.352-0.689)	<0.001
Chemotherapy	no				
	yes	0.854 (0.700-1.010)	0.065		
Radiotherapy	no				
	yes	1.458 (1.100-1.932)	0.009		

### Development and Validation of Cancer Cachexia Diagnostic Nomogram in Lung Cancer Patients

**Figure 2:**
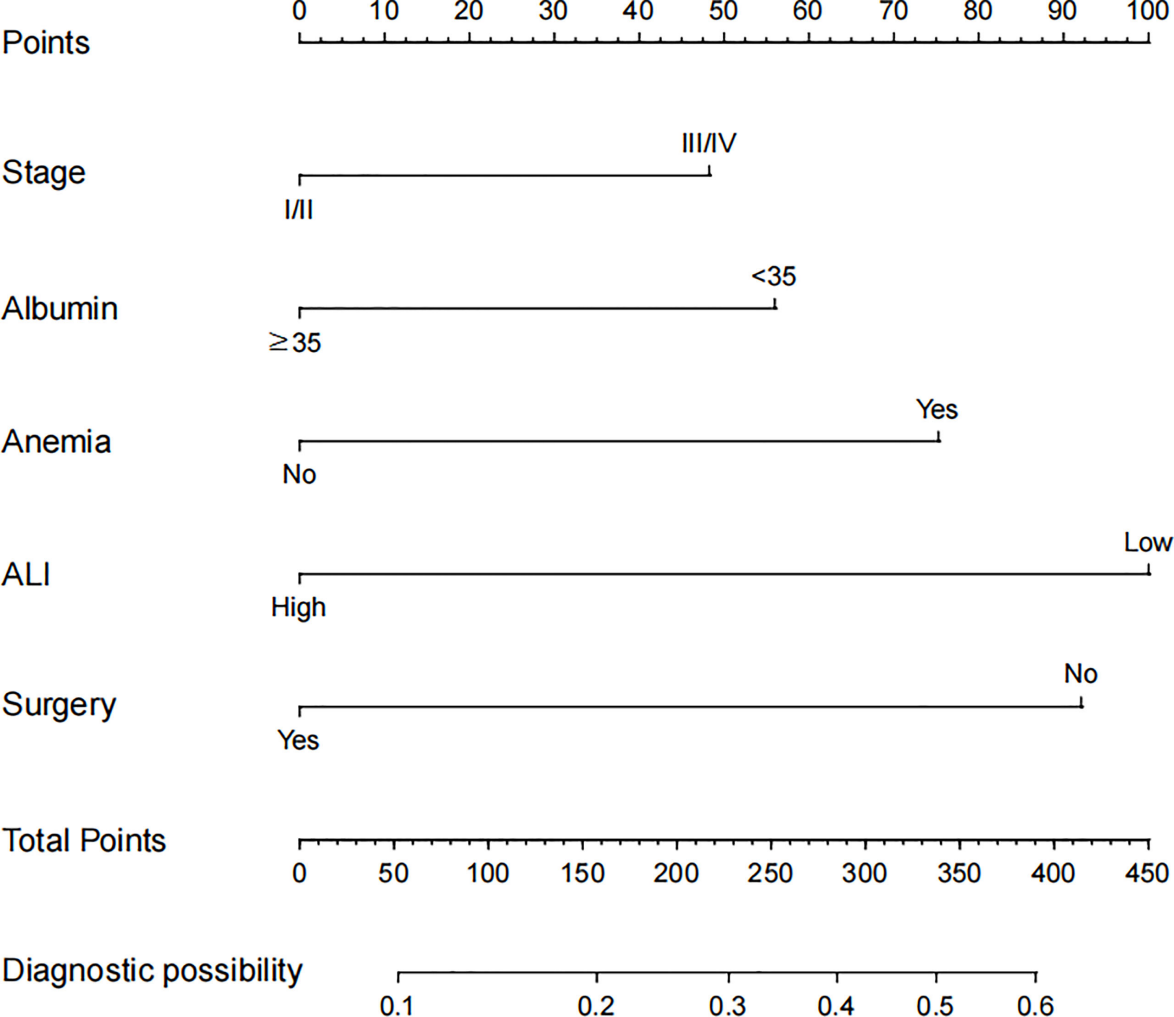
Nomogram for predicting the risk of cancer cachexia in lung cancer patients.

**Figure 3:**
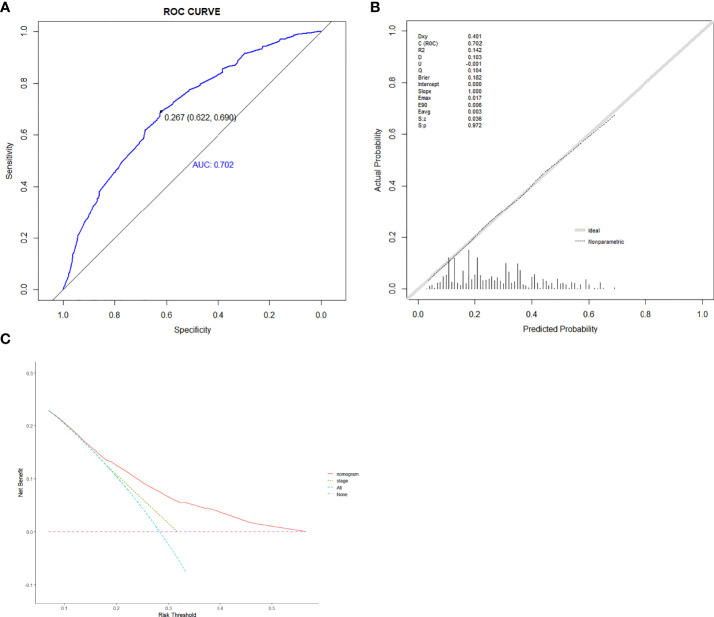
The receiver operating characteristic curve **(A)**, calibration curve **(B)**, and decision curve analysis **(C)** of the diagnostic nomogram in training set.

**Figure 4:**
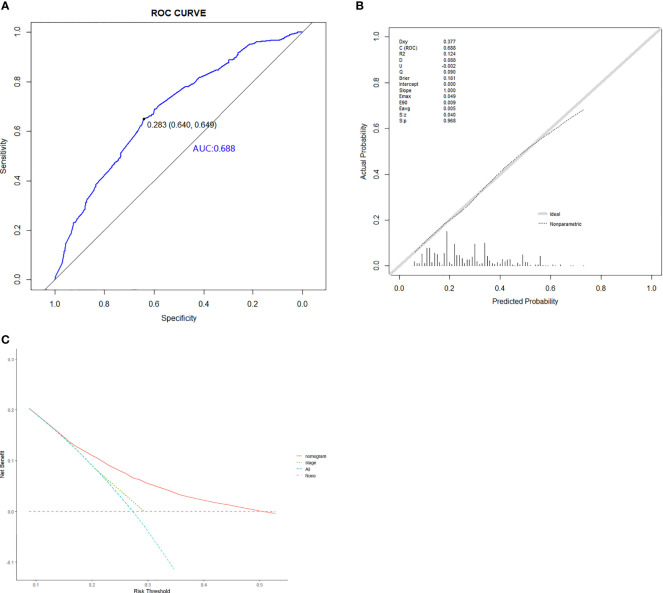
The ROC curve **(A)**, calibration curve **(B)**, and decision curve analysis **(C)** of the diagnostic nomogram in validation set.

Based on the abovementioned independent predictors, we constructed a diagnostic nomogram to evaluate the risk of cancer cachexia in patients with lung cancer (**Figure 2**). The scores of advanced stage, hypoalbuminemia, anemia, no surgical treatment, and low ALI treatment were approximately 50, 55, 75, 92.5, and 100, respectively. According to the sum of the scores, we could obtain the total score of patients and the corresponding incidence probability of cancer cachexia. Meanwhile, we used the ROC curve, calibration curve, and DCA to evaluate the model. The AUC values of the model in the training set, as well as the validation set, were 0.70 and 0.69, respectively (**Figures 3A**, **4A**). The calibration curves of training verification sets conformed to the standard curve (**Figures 3B**, **4B**). The nomogram also revealed better clinical decision-making efficiency in the DCA of the two data groups. When compared with the prediction of cachexia in patients with lung cancer by tumor stage alone, the diagnostic nomogram provided a higher net benefit (**Figures 3C**, **4C**).

### Prognostic Factors in Patients With Lung Cancer Cachexia

We first assessed all patients by the K–M analysis. The median OS of 1126 lung cancer patients with cachexia was 17.90 months (95%CI: 16.16–19.64), while that of 2896 lung cancer patients without cachexia was 32.30 months (95%CI: 29.46–35.12). As shown in **Figure S4**, the occurrence of cachexia (depicted as a blue curve) in patients with lung cancer significantly reduced the survival time of patients and increased the disease burden of patients (P < 0.001). Second, in order to comprehensively evaluate the factors that may have affected the prognosis of patients, in addition to the abovementioned factors, we included the BMI, PG-SGA, KPS, and NRS 2002 within 48 h of admission. Univariate COX analysis revealed that age, sex, BMI, smoke, stage, albumin, ALI, anemia, KPS, NRS 2002, surgery, and radiotherapy all affected the prognosis of patients with lung cancer cachexia. When these factors were included in the multivariate COX analyses, we noted that sex, stage, albumin, ALI, surgery, and KPS score served as independent prognostic factors for patients with lung cancer cachexia (**Table 3**). Male (P < 0.001) and advanced stage (III/IV, P < 0.001) are the risk factors of prognosis, while high albumin (>35 g/L, P = 0.004), high ALI (>34.93, P = 0.003), high KPS (≥70, P < 0.001), and surgery (P = 0.006) served as the protective factors of prognosis.

**Table 3:** Cox regression analysis of lung cancer patients with cachexia.

		Univariate analysis		Multivariate analysis	
		HR (95%CI)	P	HR (95%CI)	P
Age (years)	≤65				
	>65	1.299 (1.084-1.555)	0.004		
Sex	famale				
	male	1.503 (1.239-1.825)	<0.001	1.425 (1.172-1.733)	<0.001
BMI	underweight	1.341 (1.085-1.658)	0.007		
	normal		0.056		
	overweight	1.099 (0.860-1.405)	0.450		
	obesity	0.929 (0.460-1.876)	0.837		
Diabetes	no				
	yes	1.013 (0.758-1.354)	0.928		
Hypertension	no				
	yes	1.043 (0.831-1.309)	0.716		
Coronary heart disease	no				
	yes	1.016 (0.656-1.572)	0.945		
Family history	no				
	yes	0.930 (0.729-1.187)	0.561		
Smoke	no				
	yes	1.366 (1.134-1.644)	0.001		
Drinking	no				
	yes	1.157 (0.949-1.410)	0.149		
Stage	1/2				
	3/4	3.219 (2.363-4.385)	<0.001	2.628 (1.919-3.600)	<0.001
Scr	low	1.068 (0.889-1.284)	0.480		
	normal		0.296		
	high	1.313 (0.928-1.858)	0.124		
Albumin (g/L)	<35				
	≥35	0.599 (0.500-0.718)	<0.001	0.755 (0.623-0.914)	0.004
ALI	low				
	high	0.571 (0.463-0.704)	<0.001	0.715 (0.574-0.891)	0.003
Platelet (×10^9^/L)	<100				
	≥100	1.119 (0.786-1.595)	0.532		
Anemia	no				
	yes	1.533 (1.285-1.829)	<0.001		
KPS	<70				
	≥70	0.537 (0.422-0.683)	<0.001	0.613 (0.479-0.785)	<0.001
PG SGA	low				
	high	1.175 (0.677-2.042)	0.566		
NRS 2002	low				
	high	1.221 (1.017-1.465)	0.032		
Surgery	no				
	yes	0.497 (0.327-0.756)	0.001	0.547 (0.357-0.838)	0.006
Chemotherapy	no				
	yes	0.866 (0.725-1.035)	0.114		
Radiotherapy	no				
	yes	1.368 (1.040-1.799)	0.025		

### Development and Validation of Prognostic Nomogram in Lung Cancer Patients With Cachexia

**Figure 5:**
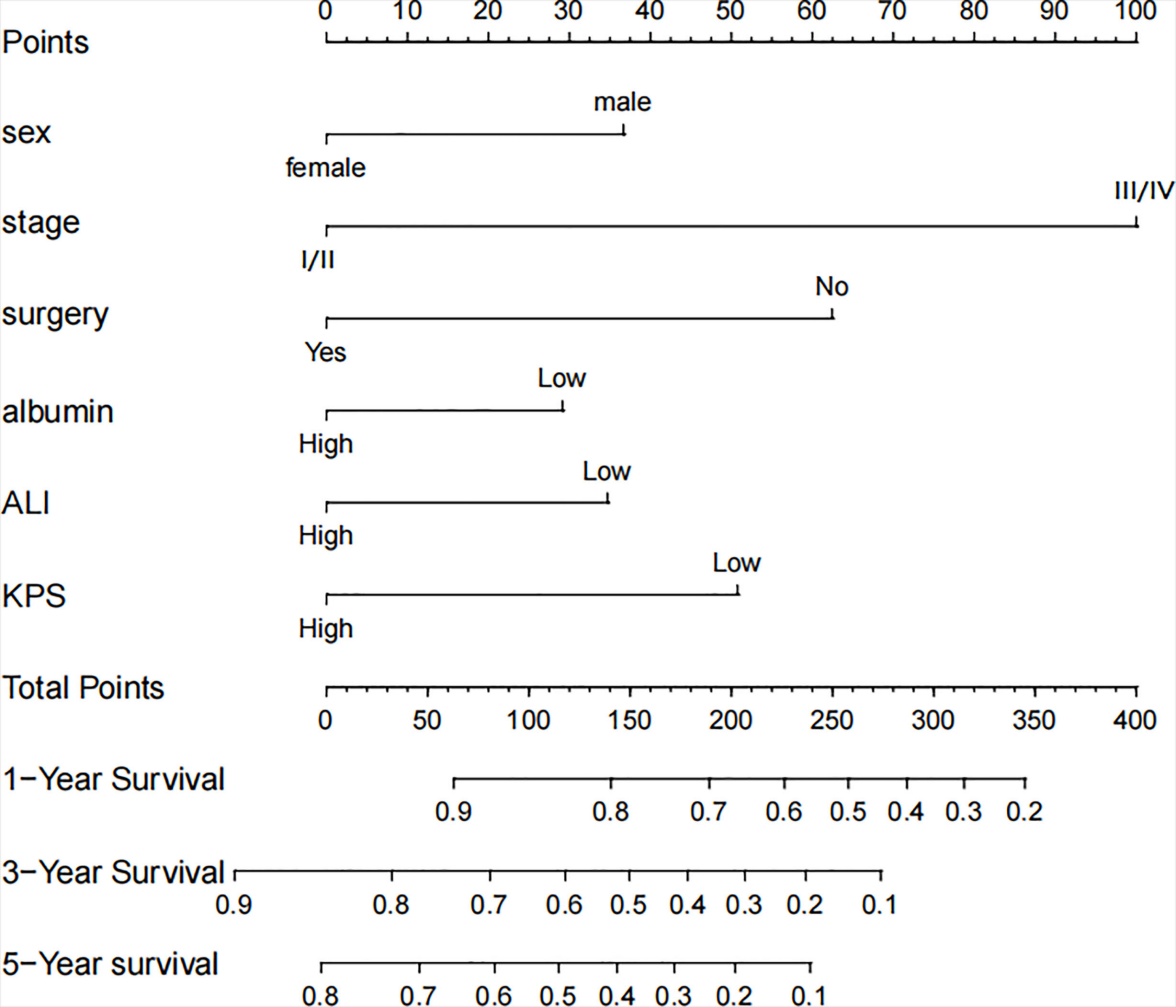
Nomogram for predicting the prognosis of lung cancer patients with cachexia.

**Figure 6:**
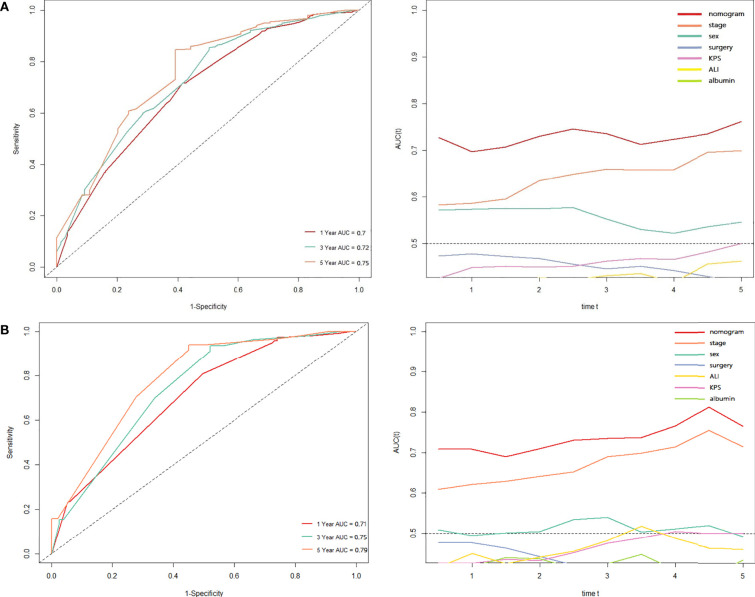
The time ROC curve of the prognostic nomogram in training **(A)** and validation sets **(B)**.

**Figure 7:**
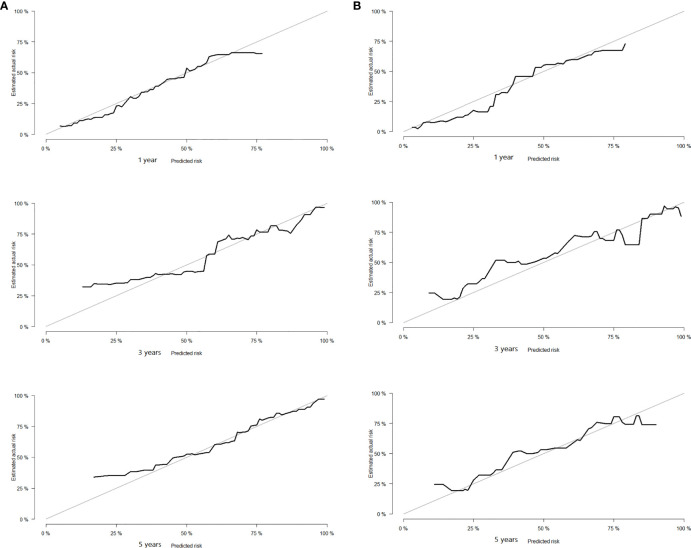
The calibration curve of the prognostic nomogram in training **(A)** and validation sets **(B)**.

**Figure 8:**
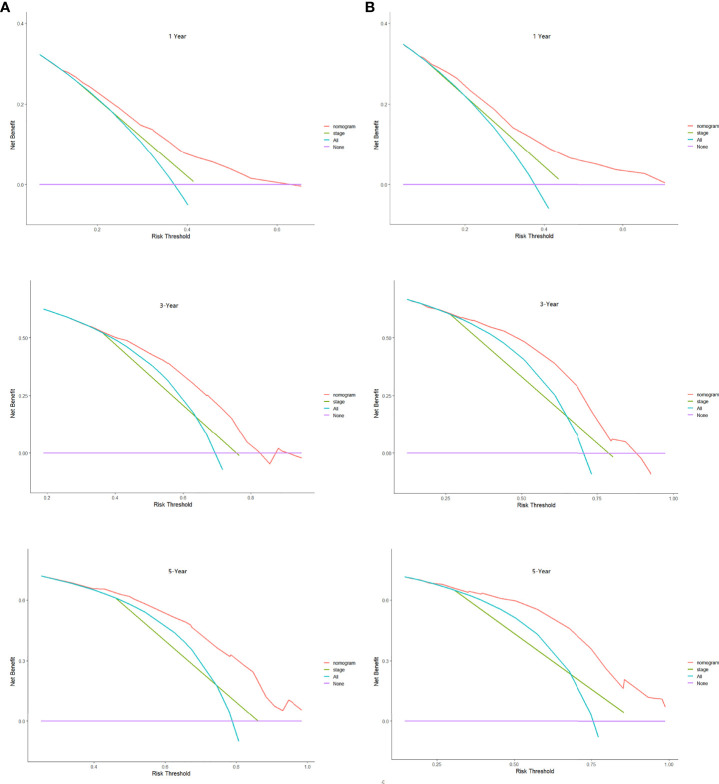
The decision curve analysis of the prognostic nomogram in training **(A)** and validation sets **(B)**.

Based on the results of Cox regression analysis, we constructed a nomogram that could predict the 1-, 3-, and 5-year survival probability of patients with lung cancer cachexia (**Figure 5**). Male gender, stage III/IV, no surgical treatment, low albumin level, low ALI, and low KPS accounted for approximately 35, 100, 62.5, 30, 35, and 50 points, respectively. Similarly, considering the sum of patients’ scores, we obtained the total score of patients and the corresponding survival probability. In the training set, the AUCs of 1-, 3-, and 5-year of nomogram were 0.70, 0.72, and 0.75, respectively, and the time ROC curve indicated that the AUC of this nomogram at any time point in 5 years was greater than that of any single factor, reflecting the good prediction efficiency of our prognostic model (**Figure 6A**). The slopes of 1-, 3-, and 5-year calibration curves of the nomogram were also close to 1 (**Figure 7A**). When compared with relying solely on the tumor stage to judge the prognosis of patients with lung cancer cachexia, DCA suggested that the nomogram had a good clinical application value (**Figure 8A**). Similarly, in the validation set, the AUC of the nomogram in 1-, 3-, and 5-year reached 0.71, 0.75, and 0.79, respectively (**Figure 6B**). The calibration curves at three time-points of nomogram and DCA also revealed similar levels (**Figures 7B**, **8B**).

### Subgroup Analyses

In order to further evaluate the accuracy and practicability of the nomograms among the subgroups, we divided the patients into two groups, early patients (stage I/II) and advanced patients (stage III/IV), and tested the accuracy and clinical decision-making efficiency of our diagnostic and prognostic nomogram among the subgroups. These results indicated that the AUCs of the diagnostic nomogram were 0.633 and 0.665 in the early and advanced patients, respectively (**Figures S5A**, **S6A**). The analysis of the calibration curve and DCA also demonstrated a good fit and a good clinical decision-making ability (**Figures S5**, **S6**). The time ROC revealed that the prognostic nomogram had high AUC values. In early patients, the AUC values of 1-, 3-, and 5 years were 0.64, 0.66, and 0.65, respectively (**Figure S7A**). In advanced patients, the AUC of 1-, 3-, and 5-years were approximately 0.65 (**Figure S8A**). The analysis of the calibration curve and DCA also indicated that the prediction efficiency of the prognosis model was extremely good (**Figures S7**, **S8**).

## Discussion

Cancer cachexia is considered a multifactorial syndrome. Although its specific pathogenesis is not clear, its high incidence rate demands attention. Peterson et al. reported that the incidence rate of cancer cachexia was approximately 25–80% (10), while the incidence of cachexia in lung cancer patients reached 27.9%. Therefore, the study on cachexia of lung cancer is particularly important. To the best of our knowledge, this is currently the largest real-world clinical study conducted on lung cancer cachexia. In this study, we analyzed the risk factors of cachexia in lung cancer patients as well as the prognostic factors of lung cancer patients with cachexia and constructed two accurate and practical nomograms to facilitate active identification of patients with cachexia and predict the prognosis of patients with cachexia.

As is well-known, the diagnosis of cachexia requires a detailed understanding of the patient’s past weight changes, BMI changes, or skeletal muscle changes, albeit several patients do not often pay attention to their weight and it is not convenient to detect their muscle mass on a timely basis, which challenges the identification and diagnosis procedures of cachexia. Although some recent studies have constructed a unique cancer cachexia diagnosis model based on the metabonomic characteristics of patients, these projects are expensive and inconvenient to obtain (11). The results of the present study compensate for the deficiency in the definition of cachexia and past studies. We have included the data of the patient’s demographic characteristics, medical history, stage, treatment methods, laboratory data (such as inflammatory and nutritional indicators), and scale scores. These items are very easy to obtain for application in a clinical study.

Notably, although we comprehensively analyzed the characteristics of each patient in four aspects (clinical characteristics, personal history, tumor characteristics, laboratory indices), not all factors were equally important. We found some primary factors, which were based on tumor characteristics, inflammation and nutrition. Regardless of the diagnostic model or prognosis model used, the tumor stage (OR: 1.450, 95% CI: 1.152–1.825, HR: 2.628, 95% CI: 1.919–3.600), surgery (OR: 0.492, 95% CI: 0.352–0.689, HR: 0.547, 95% CI: 0.357–0.838), ALI (OR: 0.463, 95% CI: 0.384–0.559, HR: 0.715, 95% CI: 0.574–0.891), and albumin (OR: 0.605, 95% CI: 0.526–0.802, HR: 0.755, 95% CI: 0.623–0.914) acted as independent diagnostic or prognostic factors.

Although we noted that cachexia could occur in patients with lung cancer at any stage, patients with advanced tumor stage were more likely to develop cancer cachexia (P < 0.001). In addition, the subgroup analysis in this study demonstrated that, although the diagnostic and prognostic nomograms showed good performance in the early and advanced-stage patients, the two nomograms in the advanced patients’ group showed a higher fitting degree, better prediction efficiency, and more stable prediction ability. Past studies have demonstrated that the high incidence of cachexia in patients with advanced cancer may be closely related to the large release of inflammatory factors in patients with advanced cancer, the disorder of insulin-like growth factor-1, and the disorder of lipid and protein metabolism caused by a long-term negative nitrogen state (19, 20). In addition, as mentioned in the review of Maccio et al., loss of appetite is a common protective behavior of the human immune system in the face of the violent proliferation of tumors in the body. Patients with advanced lung cancer often have further eating difficulties or anorexia due to the increase of tumor load, tumor compression and other phenomena, resulting in a sharp increase in the incidence rate of cachexia in patients with advanced lung cancer. Therefore, any effective antineoplastic treatment (surgery and/or chemotherapy) able to reduce the tumor burden is able to counteract the catabolic drivers and the metabolic or inflammatory changes that are involved in the pathogenesis of cachexia, and thus revert cachexia (21). This study found that the incidence of cachexia in lung cancer patients undergoing early surgery can be significantly reduced and lead to a better prognosis. Because surgical treatment itself is one of the best treatment methods to remove the primary focus, reduce tumor load, and reduce complications (22)

Different from the clinical characteristics of tumors such as tumor stage and surgery, ALI may be one of the most useful and relevant laboratory indices in clinical application. It can detect precachexia and cahcexia early from the aspect of body inflammation. Inflammation runs through the entire course of tumor development, including during tumor occurrence, development, transformation, invasion, and metastasis (23). Inflammation is an important feature of cancer as it assists in the inhibition of the host’s immune response, enhances genomic instability, destroys the tumor microenvironment, increases the risk of cancer development, affects the interaction of immune cells, and contributes to poor prognosis (24). Zhang et al. have demonstrated that the inflammatory state of a body at the baseline is an important negative prognostic biomarker in cancer cachexia patients (25), and ALI is a comprehensive index of BMI, albumin, neutrophils, and lymphocytes, thus reflecting systemic inflammation (26). Although multiple studies have demonstrated that low ALI, which represents more severe inflammation in the body, is associated with poorer prognosis in lung cancer patients (27, 28), the relationship between ALI and cachexia in lung cancer patients has not been reported in the literature. This study is the first to demonstrate that low ALI is closely related to the occurrence of lung cancer cachexia and poor prognosis. The albumin level is the most direct and sensitive expression of a body’s nutritional status. Accordingly, Xie et al. reported that the ratio of albumin to globulin is an independent prognostic factor for patients with cancer cachexia, especially for advanced patients. When compared with other malnutrition assessment tools, it demonstrates better prognostic stratification ability for patients with cancer cachexia (15). This study also confirmed the relevant views that the decrease in the albumin level can significantly affect the prognosis of patients with lung cancer cachexia and validated the notion that the occurrence of hypoproteinemia may be related to the occurrence of cachexia in patients with lung cancer.

The best treatment for cancer cachexia depends on the disease stage (29), because, before developing into refractory cachexia, the patient’s condition can be reversed through the use of effective drugs (30), nutritional support (31), exercise (32), and psychosocial support (33). Therefore, it is extremely important to identify, monitor, and treat cancer cachexia at the earliest; hence our research is of great significance. Although our study is a large-scale real-world clinical assessment involving more than 40 clinical centers across China, the study has some limitations. For instance, first, only Chinese patients were included in this cohort, which made the bias of genetic background, lifestyle, and dietary patterns inevitable. Second, because this is a retrospective analysis based on a cohort study, the level of evidence is low, which needs to be further verified by well-designed large-scale, multi-center, and multi-country prospective studies. Third, our study included patients of different ages and other different characteristics, who were heterogeneous. So molecular type, driver gene expression, PD-L1 expression, and tumor mutation burden are particularly important for the prognosis of lung cancer patients. However, unfortunately, this information that may enhance the accuracy of a model and treatment personalization was not included in the study. Nevertheless, we intend to undertake this research direction in the future.

## Conclusion

Our study findings validated that lung cancer cachexia would add additional economic burden and contribute to a poor prognosis. Patients with advanced diseases, low levels of albumin and ALI, anemia, and primary focus without any surgical treatment have a higher risk of cancer cachexia. Moreover, the prognosis of lung cancer cachexia patients with advanced disease, male gender, low albumin level, low ALI, low KPS score, and a primary focus without surgical treatment is worse. Meanwhile, we also established two convenient and individualized nomograms for the diagnosis, screening, and prognosis prediction of lung cancer cachexia.

## Data Availability Statement

The raw data supporting the conclusions of this article will be made available by the authors, without undue reservation.

## Ethics Statement

The studies involving human participants were reviewed and approved by Ethics Committee of Daping Hospital, Army Medical center of PLA. The patients/participants provided their written informed consent to participate in this study.

## Author Contributions

H-PS formulated the research plan, supervised the completion of the research and provided financial support. C-AL and QZ wrote the manuscript. C-AL and L-YS participated in the completion of pictures and tables. G-TR, H-LX, TL, and MT collected the data, while XZ, MY, C-LH and K-PZ participated in the discussion and interpretation of the results. All authors contributed to the article and approved the submitted version.

## Funding

This work was supported by the National Key Research and Development Program to H-PS (No. 2017YFC1309200).

## Conflict of Interest

The authors declare that the research was conducted in the absence of any commercial or financial relationships that could be construed as a potential conflict of interest.

## Publisher’s Note

All claims expressed in this article are solely those of the authors and do not necessarily represent those of their affiliated organizations, or those of the publisher, the editors and the reviewers. Any product that may be evaluated in this article, or claim that may be made by its manufacturer, is not guaranteed or endorsed by the publisher.
