# Supplementary material for: Novel Diagnostic and Prognostic Tools for Lung Cancer Cachexia: Based on Nutritional and Inflammatory Status
Source: Front Oncol. 2022 Jul 11;12:890745. doi: 10.3389/fonc.2022.890745 (PMC9309732; doi:10.3389/fonc.2022.890745)
Supplement: Supplementary file 1 [file DataSheet_1.docx]

Fig.S1 Difference in hospitalization expenses of lung cancer patients with or without cancer cachexia


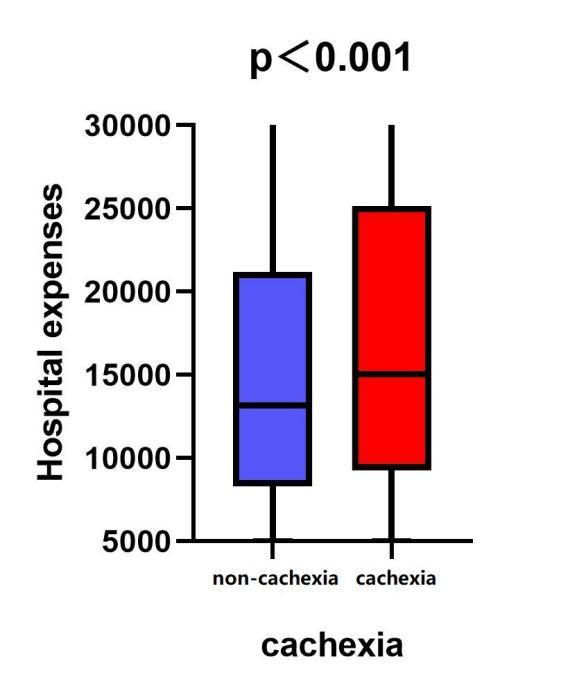


Fig.S2 The association between ALI (continuous) and hazard ratio of overall survival. (Splines is adjusted by sex, age, BMI, Tumor stage, smoke, drinking, radiotherapy, chemotherapy, surgery, albumin level, Scr, anemia, platelet count, KPS score, NRS2002 score, and PG SGA score.


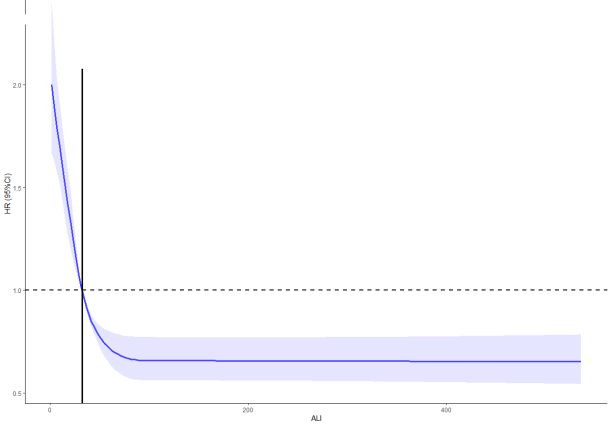


Fig.S3 Multivariable logistic regression analysis of factors associated with cancer cachexia.


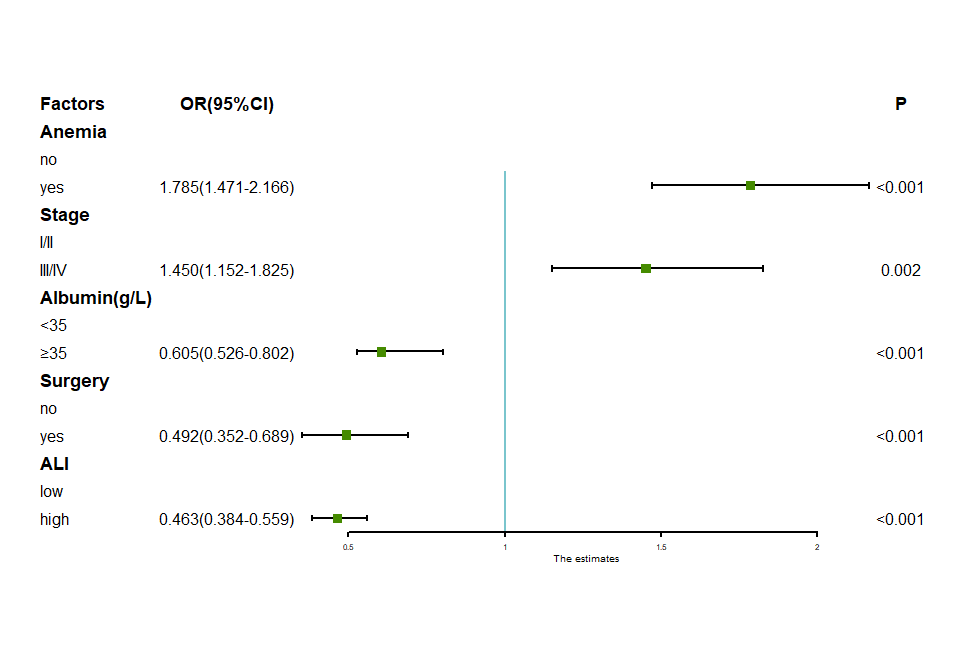
Fig.S4 Kaplan-Meier curves of overall survival for patients stratified by the presence or absence of cancer cachexia.


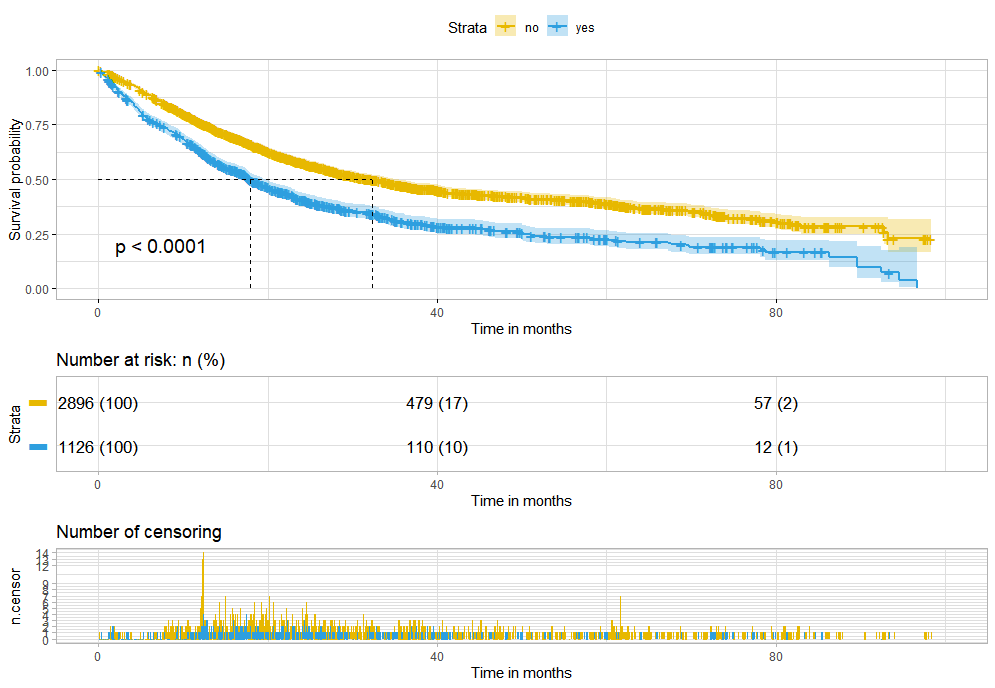


Fig.S5 ROC curve (A), calibration curve (B) and DCA (C) of diagnostic nomogram in the early stage patients subgroup.

| A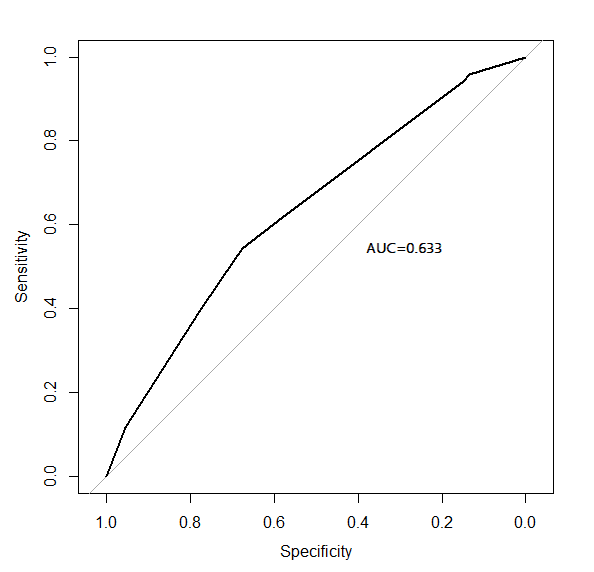 | B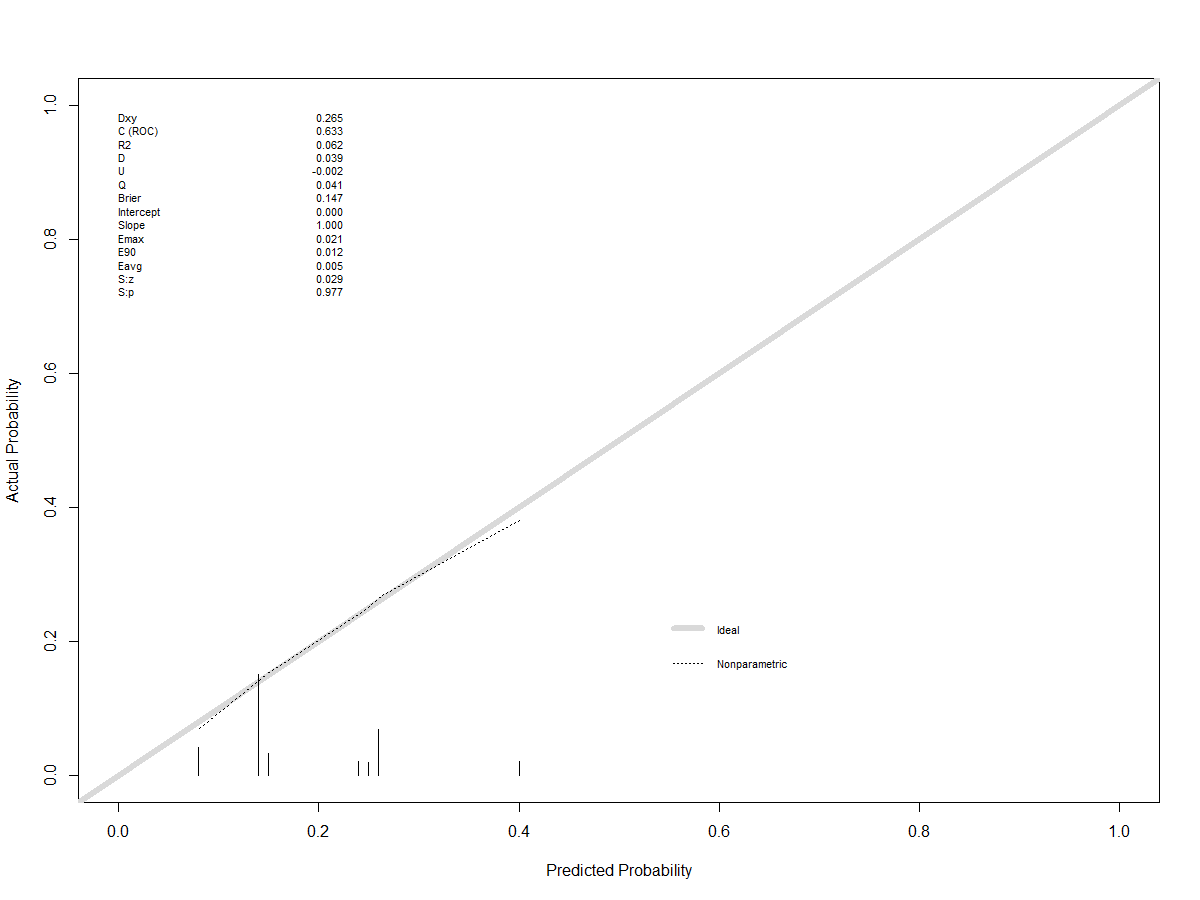 |
| --- | --- |
| C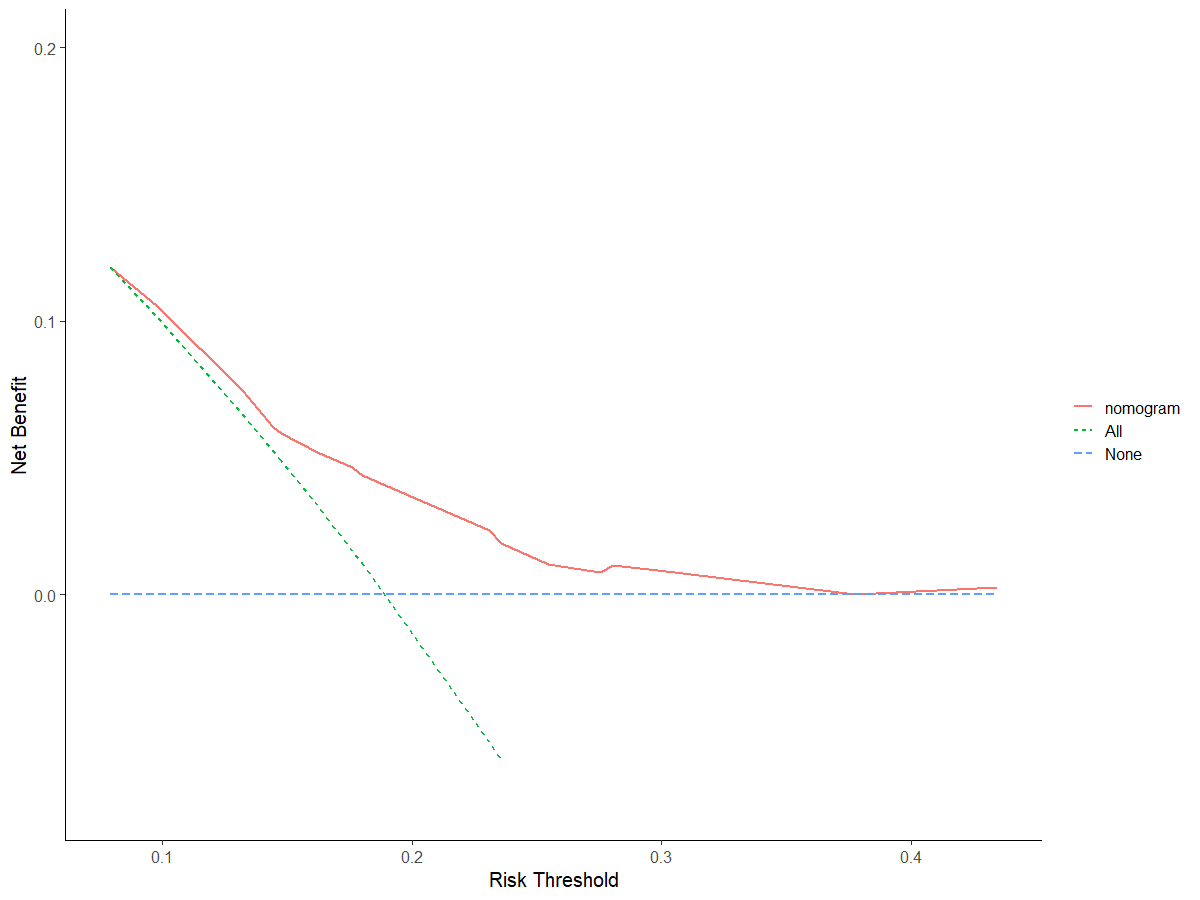 |  |

Fig.S6 ROC curve (A), calibration curve (B) and DCA (C) of diagnostic nomogram in the advanced stage patients subgroup.

| A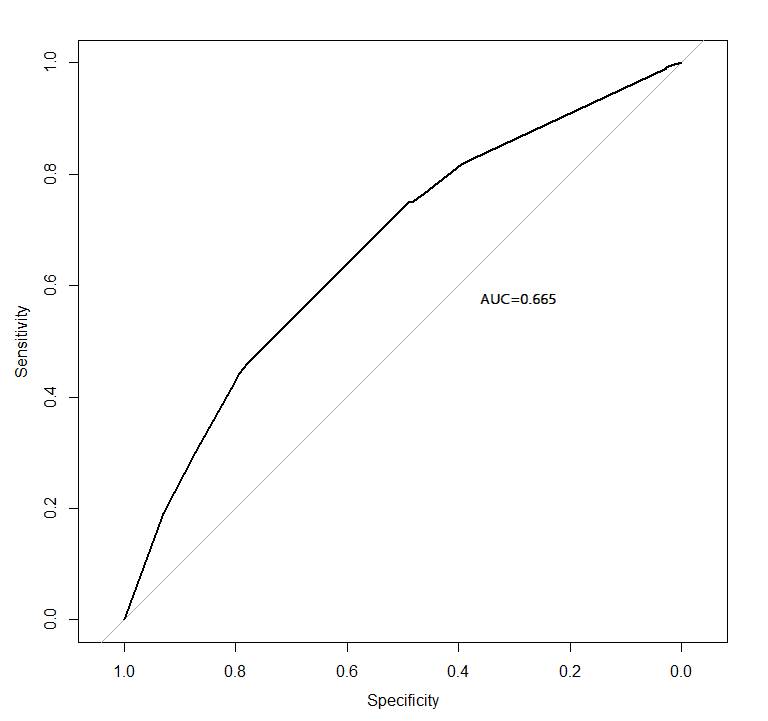 | B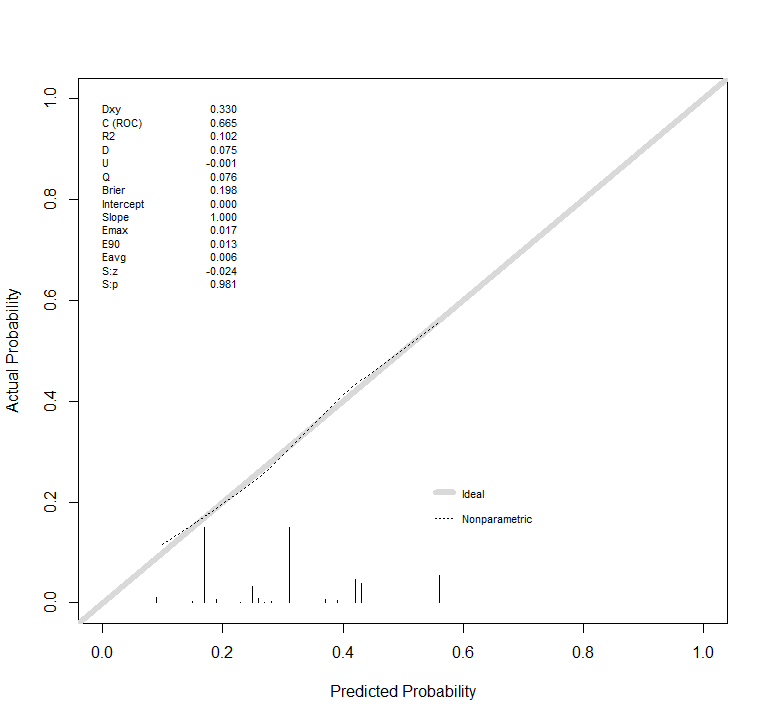 |
| --- | --- |
| C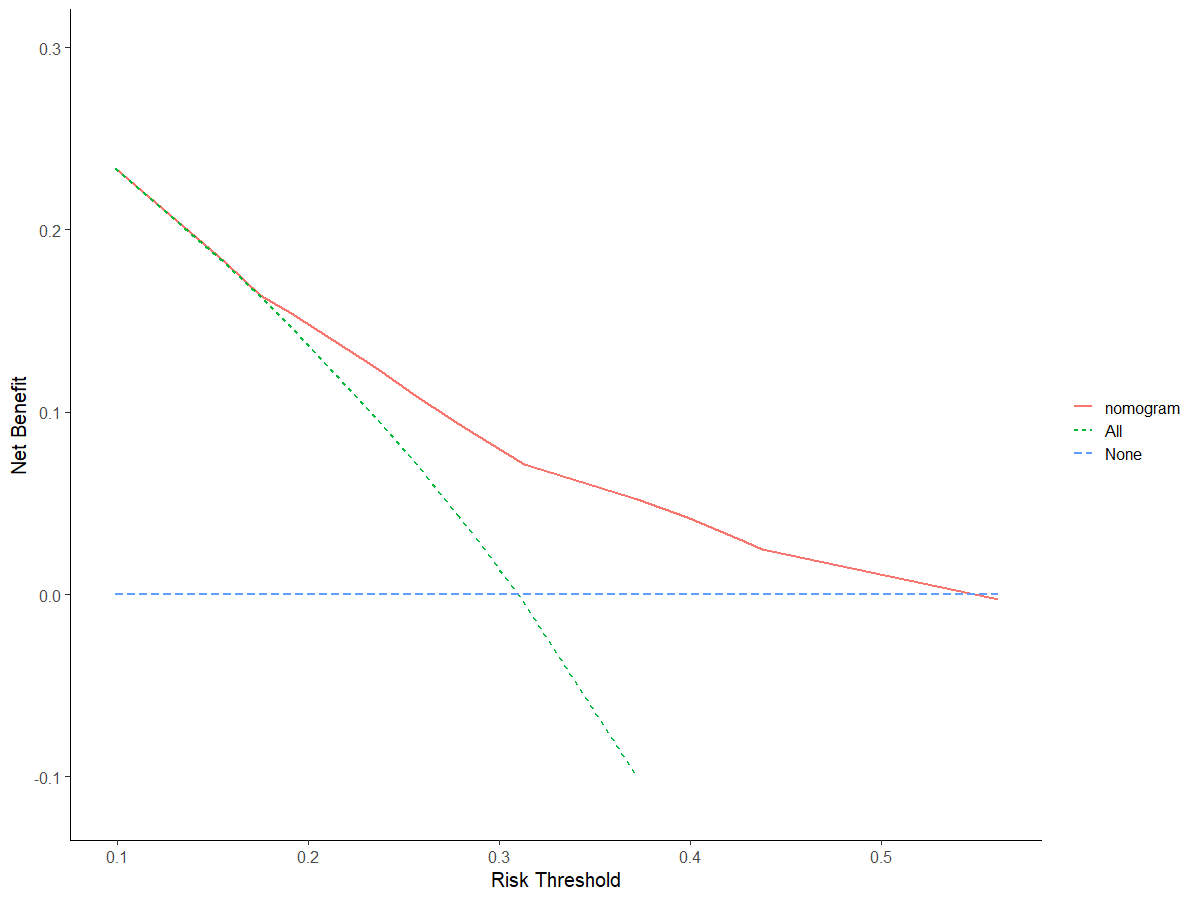 |  |

Fig.S7 The time ROC curve (A), calibration curve (B) and DCA (C) of prognostic nomogram in the early stage patients subgroup.

| A  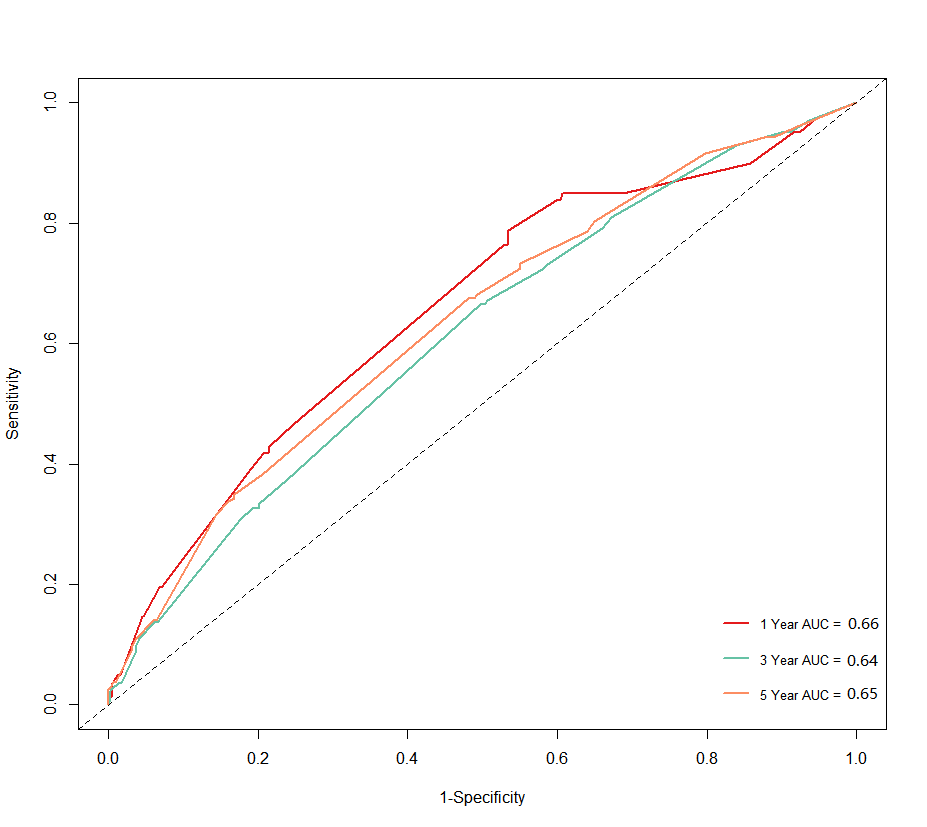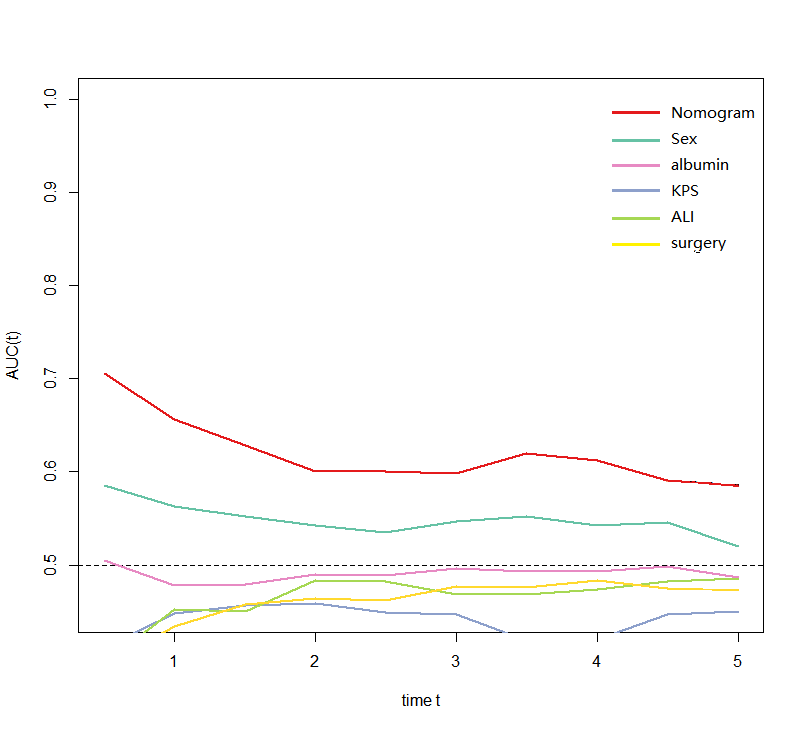 |  |
| --- | --- |
| B  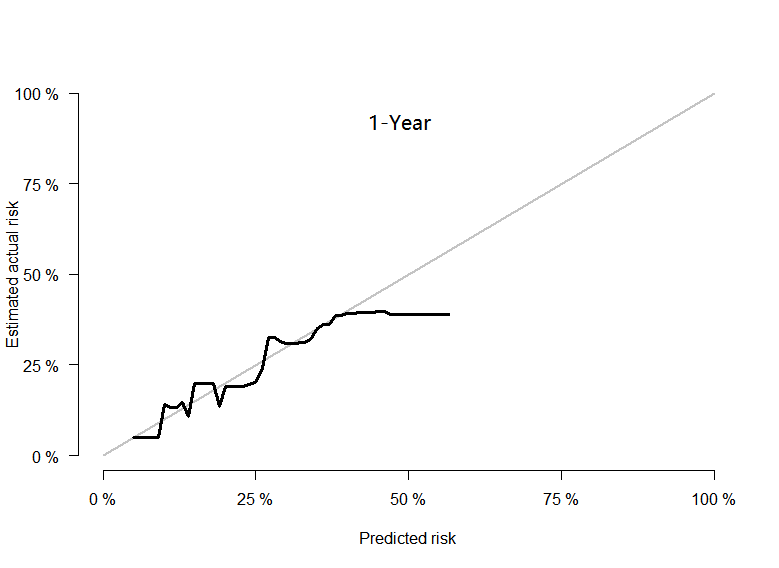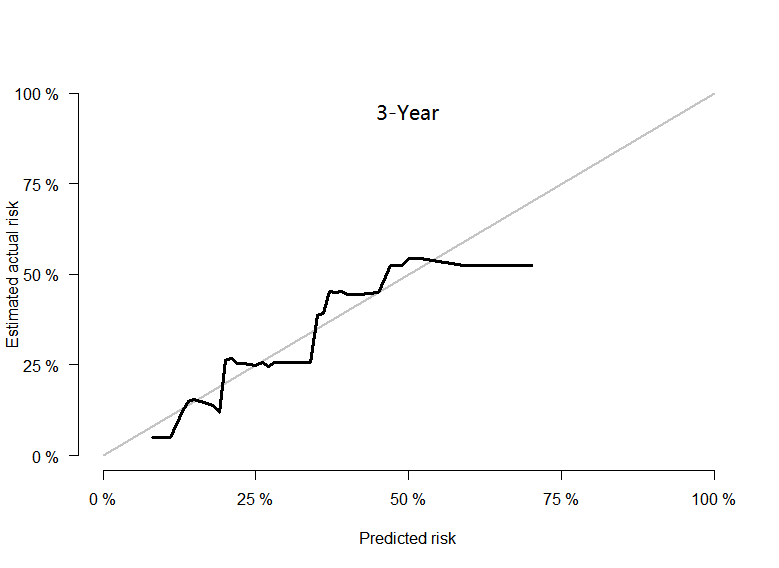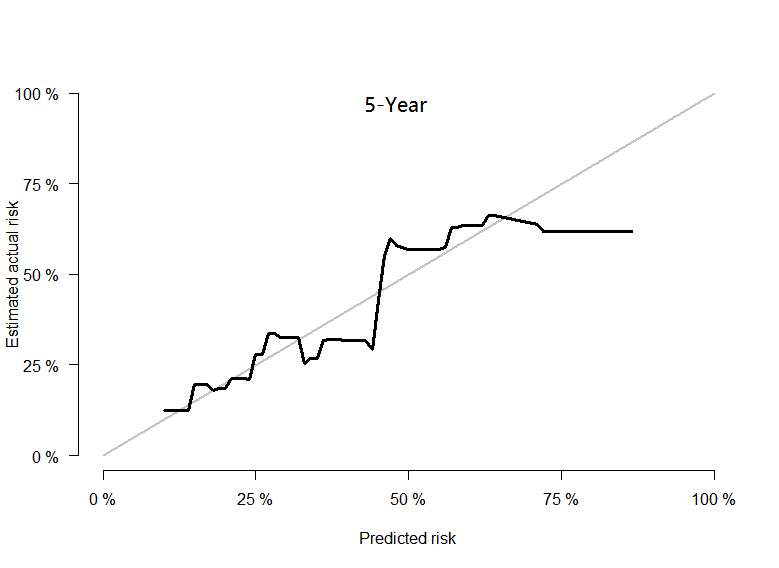 | |
| C  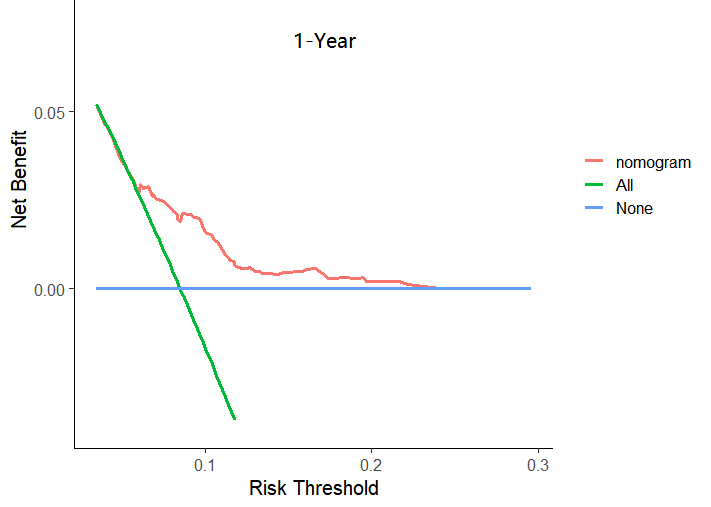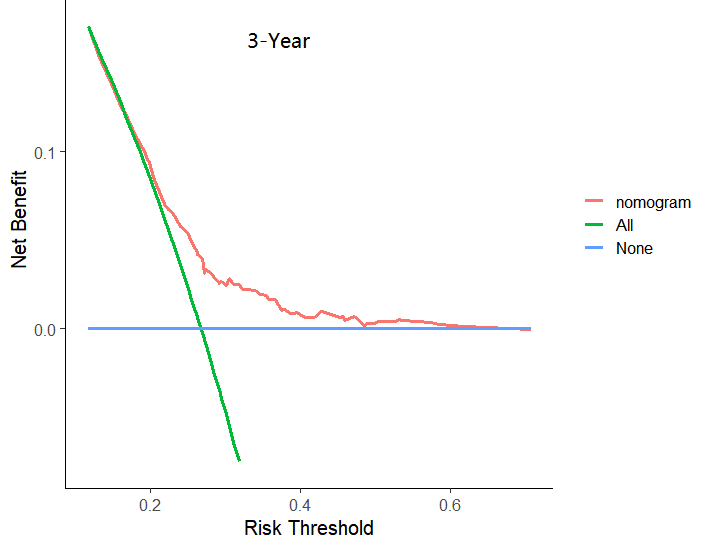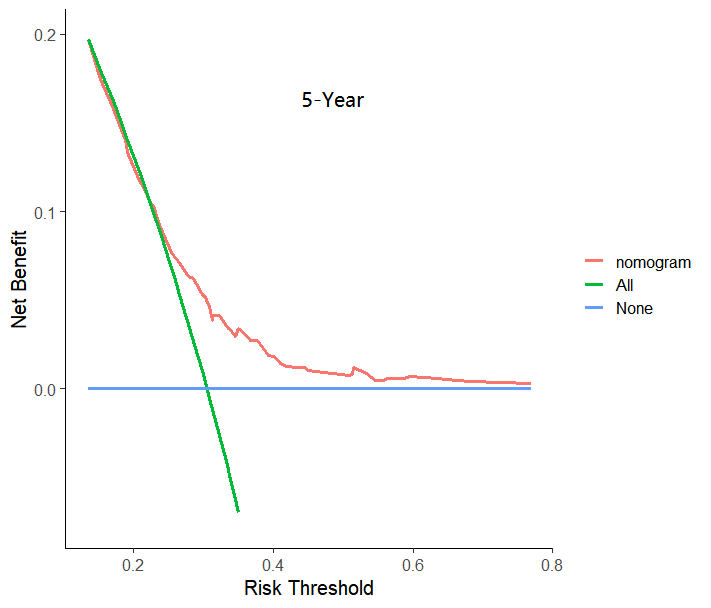 | |

Fig.S8 The time ROC curve (A), calibration curve (B) and DCA (C) of prognostic nomogram in the advanced stage patients subgroup.

| A  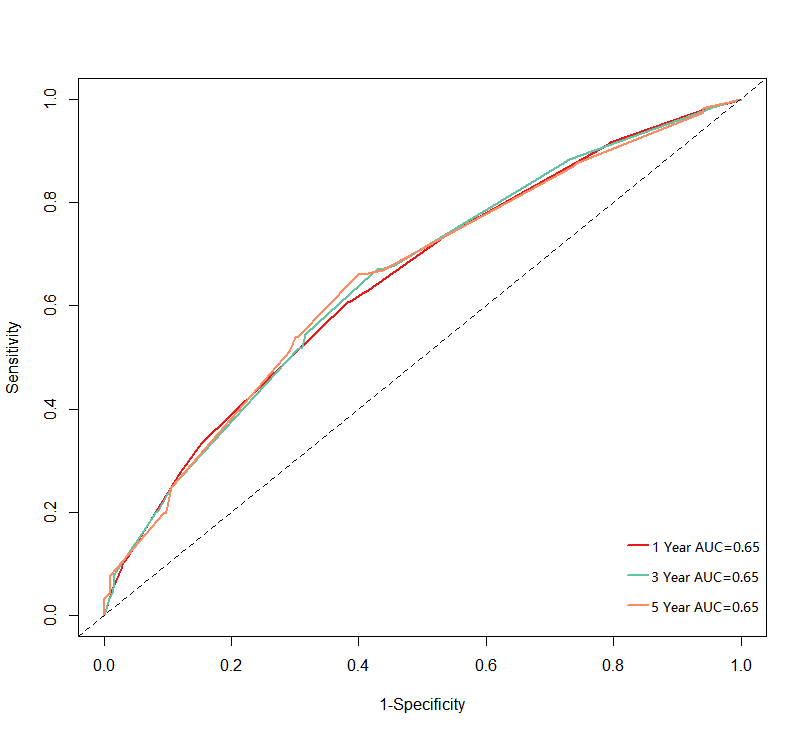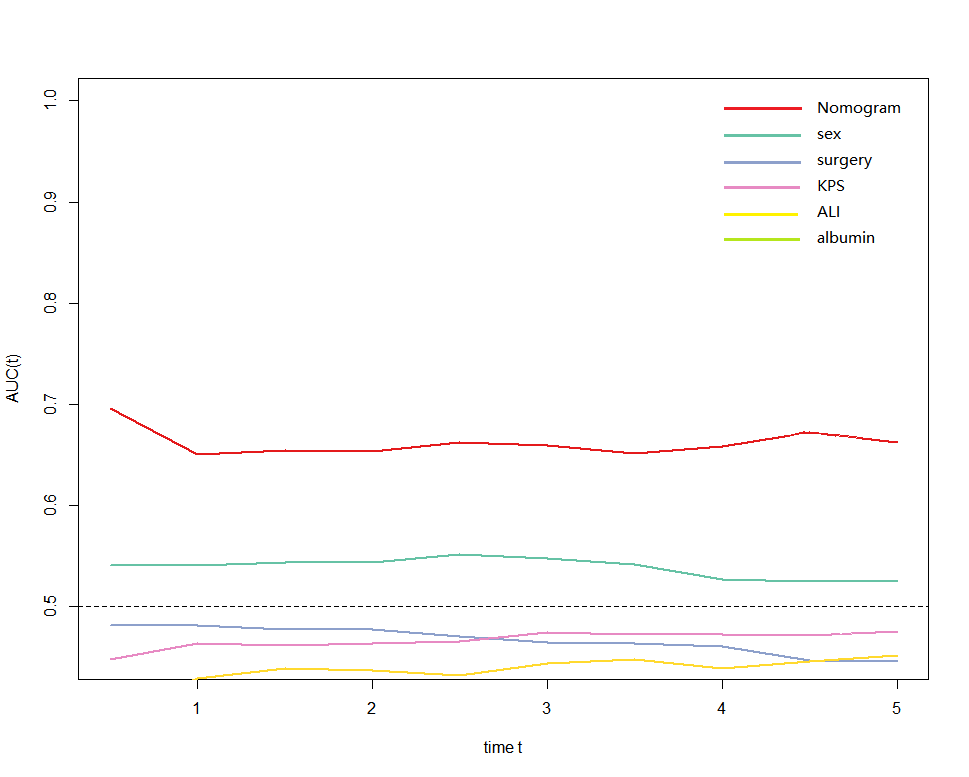 |  |
| --- | --- |
| B  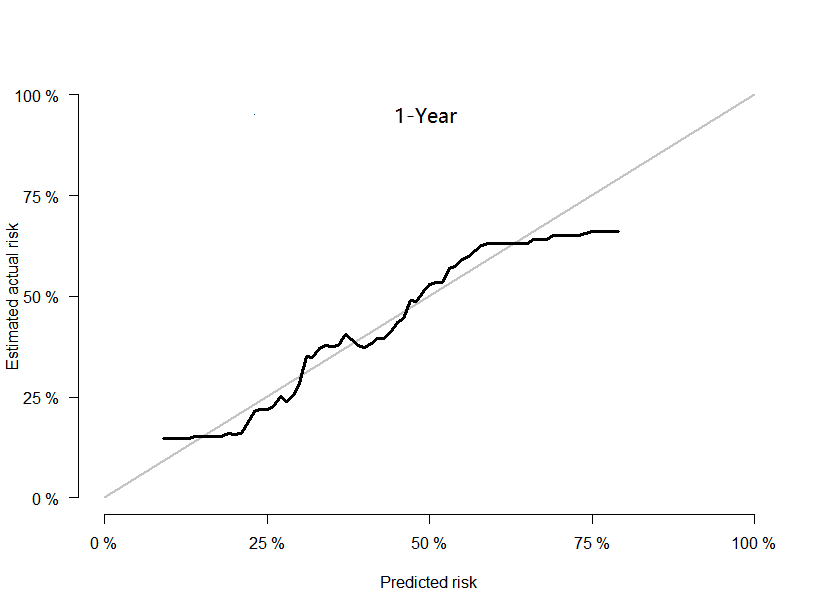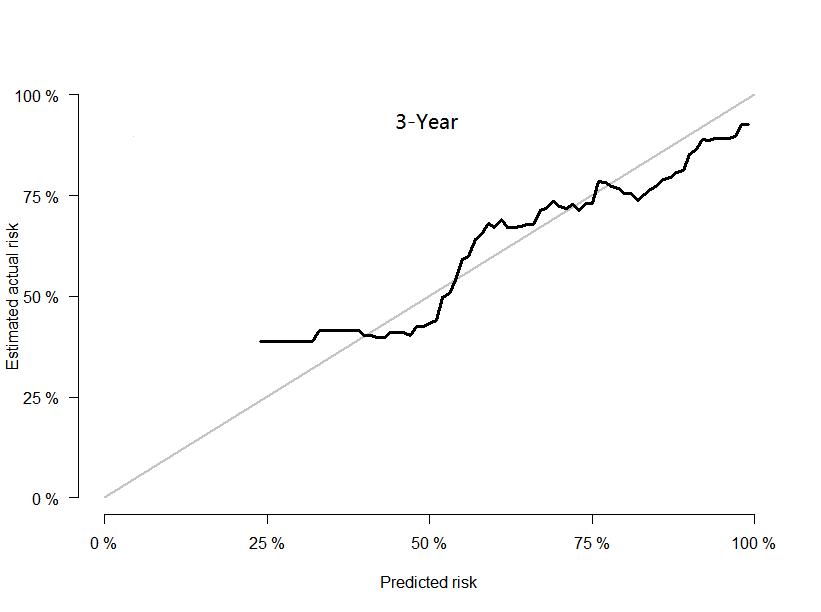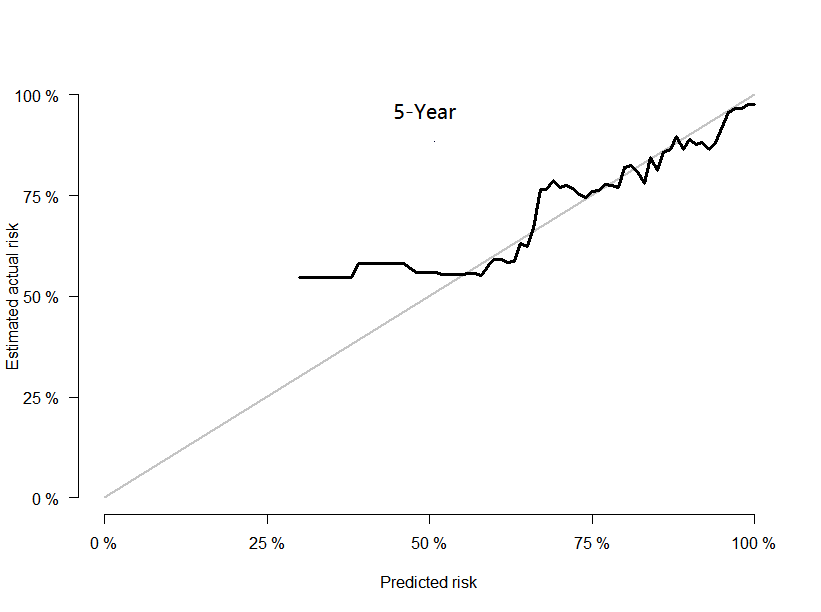 | |
| C  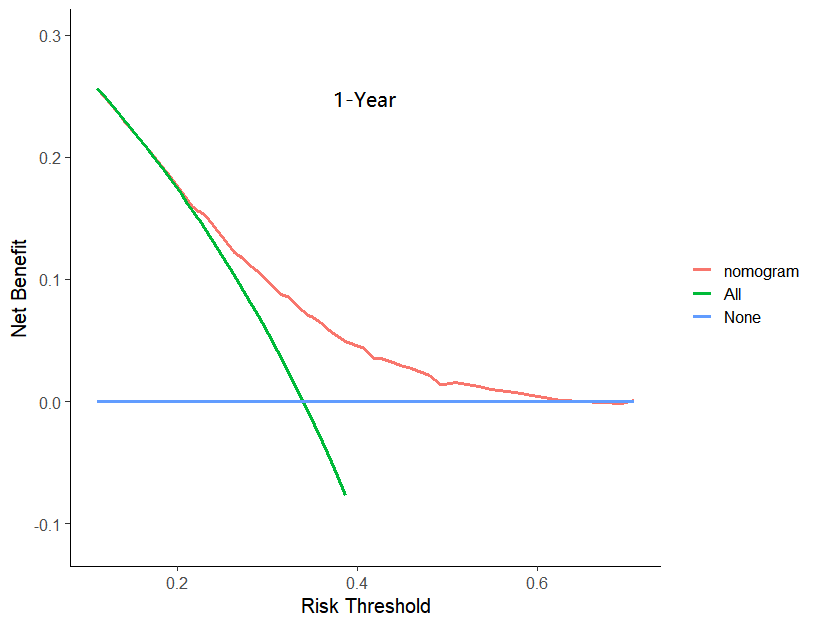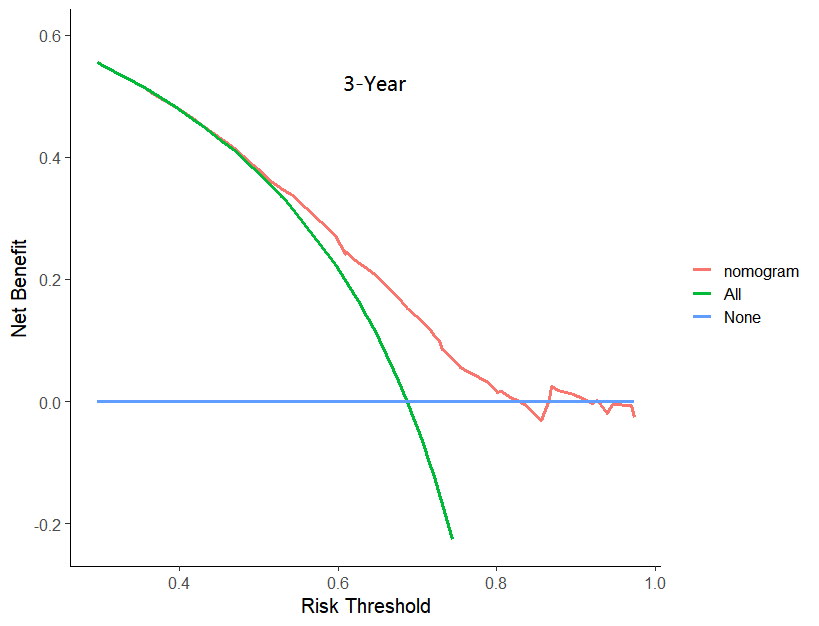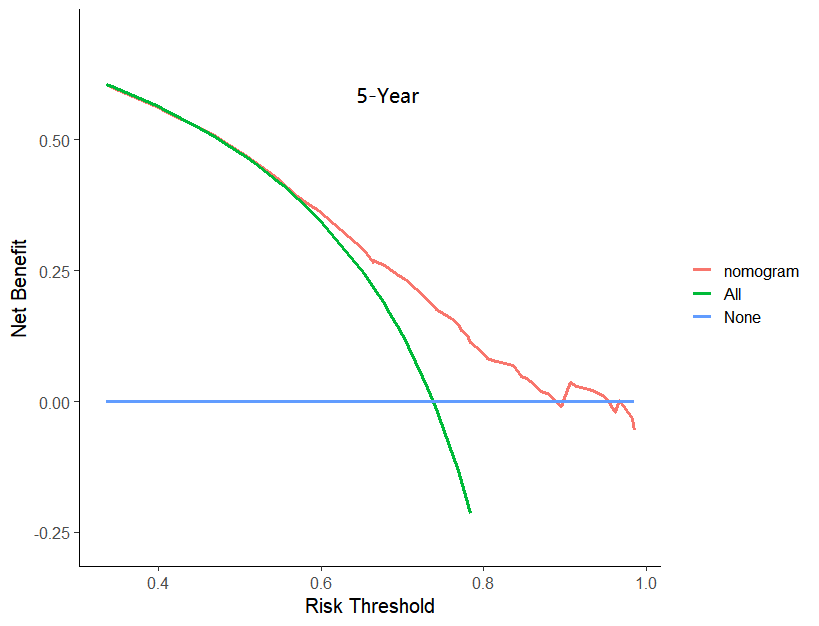 | |
